# Preparation and application of porcine broadly neutralizing monoclonal antibodies in an immunoassay for efficiently detecting neutralizing antibodies against foot-and-mouth disease virus serotype O

**DOI:** 10.1128/spectrum.02234-24

**Published:** 2025-01-08

**Authors:** Yimei Cao, Fengjuan Li, Xiangchuan Xing, Huiyan Zhang, QiongQiong Zhao, Pu Sun, Yuanfang Fu, Pinghua Li, Xueqing Ma, Jing Zhang, Zhixun Zhao, Hong yuan, Jian Wang, Tao Wang, Huifang Bao, Xingwen Bai, Dong Li, Qiang Zhang, Kun Li, Zengjun Lu

**Affiliations:** 1 State Key Laboratory for Animal Disease Control and Prevention, College of Veterinary Medicine, Lanzhou University, National Foot-and-Mouth Diseases Reference Laboratory, Lanzhou Veterinary Research Institute, Chinese Academy of Agricultural Sciences, Lanzhou, China; Regional Centre for Biotechnology, Faridabad, Haryana, India

**Keywords:** foot-and-mouth disease virus, porcine broadly neutralizing monoclonal antibody, competitive ELISA, neutralizing antibody, serotype O

## Abstract

Neutralizing antibodies provide vital protection against foot-and-mouth disease virus (FMDV). The virus neutralization test (VNT) is a gold standard method for the detection of neutralizing antibodies. However, its application is limited due to the requirement for live virus and unsuitability for large-scale serological surveillance. In this study, a porcine broadly neutralizing monoclonal antibody (PO18-10) against FMDV was obtained from the heterologous sequentially vaccinated pig using single-B-cell antibody technology. A competitive enzyme-linked immunosorbent assay (C-ELISA) for detecting neutralizing antibodies against FMDV serotype O was developed using biotinylated PO18-10 as a detector antibody. The sensitivity and specificity of the assay were 100% and 99.55%, respectively, and the positive/negative coincidence rate with VNT was 94%, suggesting that C-ELISA based on natural host-derived monoclonal antibody (mAb) could be a promising tool to detect neutralizing antibodies against FMDV serotype O and evaluate the vaccine efficacy.

**IMPORTANCE:**

Foot-and-mouth disease virus (FMDV) serotype O is one of the most prevalent serotypes in the world. The neutralizing antibody titers in primo-vaccinated animals are directly related to their level of protection against a virus challenge. The development of a safe, rapid, and accurate method for the detection of the neutralizing antibody is essential for the control and eradication of FMD. In this study, an inter-serotype broadly neutralizing monoclonal antibody PO18-10 was successfully produced using single-B-cell antibody technology from sequentially vaccinated pigs. A competitive ELISA based on this natural host-derived mAb for the detection of neutralizing antibodies against FMDV serotype O was developed and validated. The assay demonstrates high sensitivity, specificity, and coincidence rate with VNT, making it an alternative tool for confirming FMDV infection and evaluating the vaccine efficacy.

## INTRODUCTION

Foot-and-mouth disease (FMD) is a highly contagious viral disease in cloven-hoofed animals that severely affects the international trade of livestock and animal products and causes significant economic losses (1). FMD is caused by FMD virus (FMDV), which belongs to the genus *Aphthovirus* in the family *Picornaviridae*. The virus exists in seven antigenically and genetically distinct serotypes comprising O, A, Asia 1, C, and South African territory (SAT) 1, 2, and 3 as well as numerous and constantly evolving strains showing a spectrum of antigenic diversity (2, 3). FMDV serotype O is widely prevalent in China, and the circulating strains include Mya-98 lineage of Southeast Asia (SEA) topotype, Cathay topotype, and PanAsia lineage and IND2001 lineage of Middle East-South Asia (ME-SA) topotype (4).

FMD control in endemic areas mainly depends on the vaccination of susceptible livestock with inactivated vaccines. The evaluation of vaccine potency and estimation of herd immunity is necessary for the control and eradication of FMD. The standard test for FMD vaccine potency is the vaccination challenge test. However, considering animal welfare, biosafety, and economics, indirect tests, including the virus neutralization test (VNT) (5), liquid-phase-blocking enzyme-linked immunosorbent assay (ELISA) (LPB-ELISA) (6–8), and solid-phase competition ELISA (SPC-ELISA) (6, 9), have been recommended by the Office International des Epizooties (OIE). The neutralizing antibody titers in primo-vaccinated animals are closely related to their protection against virus challenge (10–12). The virus neutralization test (VNT) is considered the gold standard for detecting neutralizing antibodies of FMDV. However, the VNT requires restrictive biocontainment facilities to handle live viruses and is laborious, making large-scale serological testing difficult. Due to its safety and ease of use, LPB-ELISA has been used worldwide, but it also has some drawbacks, including a lack of antigenic stability and high false-positive reactions (13, 14). SPC-ELISA has been reported to have a higher specificity than LPB-ELISA for detecting neutralizing antibodies of FMDV (15).

To improve the assay performance, such as sensitivity and specificity, ELISAs were developed using monoclonal antibodies (mAbs) as capture and/or detection antibodies instead of polyclonal antisera. A variety of mAbs against FMDV have been produced using mouse hybridomas (16–18). Major neutralizing epitopes of FMDV identified by mouse mAbs could also be recognized by bovine antibodies (19, 20), but the relative preferences in each epitope region and fine epitope structure may differ from those of cattle. In addition, hybridoma cells have the problem of instability, and antibodies are easy to lose.

Anti-FMDV mAbs derived from natural host animals such as cattle and pigs can be generated using a single-B-cell antibody technique (21, 22). This strategy allows direct amplification of genes encoding heavy chain variable region (VH) and light chain variable region (VL) from single B cells and subsequent expression of antibodies in CHO cell lines. This method avoids complex hybridoma fusion and screening approaches and is convenient and feasible. In this study, a porcine broadly neutralizing monoclonal antibody against FMDV was produced using a combination of fluorescence-activated cell sorting (FACS) and high-throughput sequencing of porcine BCR and was used to develop a competitive ELISA for the detection of neutralizing antibodies against FMDV serotype O. The use of porcine broadly neutralizing mAb as the detecting antibody is expected to improve the accuracy of ELISA in detecting FMDV neutralizing antibodies from natural hosts (cattle, pigs, and sheep). The cutoff, sensitivity, and specificity of the competitive ELISA were determined using sera with a known status.

## MATERIALS AND METHODS

### Serum samples

#### Negative sera

A total of 220 sera were collected from healthy animals with no exposure to FMD vaccine and used to estimate the cutoff value and specificity. All negative sera were assured to be free of anti-FMDV antibodies by O- LPB-ELISA and A- LPB-ELISA (produced negative results; titer ＜ 0.6 log10).

#### Positive sera

A total of 106 sera were collected from 33 animals infected with FMDV serotype O (*n* = 33) and 73 animals vaccinated with commercial serotype O FMD-inactivated vaccines (*n* = 73) and used to estimate the cutoff value and sensitivity. The selected sera were collected at different times after infection or vaccination representing a variety of anti-FMDV antibody titers and were confirmed to be positive against FMDV serotype O by VNT. All virulent FMDV-related animal experiments were performed in the animal biosafety level 3 (ABSL-3) facility at Lanzhou Veterinary Research Institute (LVRI, Chian).

#### Field sera

A total of 150 serum samples were collected on 42 days post-primary vaccination (dpv) from 50 cattle (sera no. 1–50), 50 sheep (sera no. 51–100), and 50 pigs (sera no. 101–150), which were vaccinated with inactivated FMDV serotype O vaccines at days 0 and 21. These sera were included to evaluate the coincidence rate of the developed C-ELISA with VNT.

#### Serum samples positive for other viruses

Eight sera from porcine reproductive and respiratory syndrome virus (PRRSV)-infected swine, three sera from senecavirus A (SVA)-infected swine, three sera from classical swine fever virus (CSFV)-infected swine, three sera from peste des petits ruminants virus (PPRV)-infected ovine, and three sera from bovine viral diarrhea virus (BVDV)-infected bovines were included in this study. All sera were tested and confirmed to be free of anti-FMDV antibodies by O- LPB-ELISA and A- LPB-ELISA (produced negative results; titer ＜ 0.6 log10).

#### Serum samples positive for other serotypes of FMDV

Twenty sera positive for serotype A and 34 sera positive for serotype Asia 1 were obtained from the OIE/National Foot-and-Mouth Disease Reference Laboratory (China) and were tested to evaluate the cross-reactivity between serotypes of FMDV.

#### Serum samples from vaccinated pigs

Twenty pigs were inoculated with FMDV O/Mya/98 inactivated vaccine on days 0 and day 21, respectively. Serum samples were collected weekly and used to analyze post-vaccination kinetics of neutralizing antibody response. Sera collected at 7 and 8 weeks were also used to evaluate heterologous antibodies.

### Monoclonal antibodies

Capture antibody E32 was produced previously in our laboratory using single-B-cell isolation techniques from the peripheral blood mononuclear cells (PBMCs) of cattle that were sequentially vaccinated with three topotypes of FMDV serotype O strains and was a cross-reactive antibody against FMDV serotypes A and O with no neutralizing activity (23). Detecting antibody PO18-10 was produced from PBMCs of experimentally immunized pigs using a single-B cell antibody technique, as described previously (22). Briefly, a 3-month-old healthy pig (Sus scrofa) was primarily immunized two times with inactivated FMDV O/HN/CHA/93 vaccine (Cathay topotype, 5 µg 146S antigen formulated with ISA 201 adjuvant) with 1 month interval. On day 157 after the first immunization, the pig was further boosted with bivalent FMDV serotypes A and O vaccine that contained a mixture of four strains, O/18074 (Cathay topotype), O/Tibet/99 (ME-SA topotype), A/WH/CHA/09 (G1 subset of SEA97 lineage) and A/AF72 (A22 lineage), and corresponding 146S antigens with 5 µg each formulated with ISA 201 adjuvant. On day 5 after the final vaccination, EDTA- anticoagulated blood was sampled from the precaval veins of the pig, and PBMCs were isolated using HISTOPAQUE 1.077 (Sigma-Aldrich, USA) according to the manufacturer′s instructions.

Subsequently, the antigen-specific B cell receptors (BCRs) repertoire was constructed from the porcine PBMCs. The total B cells were enriched by negative separation to remove monocytes, NK, and T cells in PBMCs. Subsequently, the antigen-specific B cells were sorted using bait of biotinylated FMDV (O/18074 strain) 146S antigen by FACS Aria II (BD). Then, the O/18074-binding single cells were captured, and a library was constructed using the Chromium Next GEM Single Cell 5' Kit v2 (10× Genomics) (PN-1000263) according to the manufacturer’s instructions. BCR sequence repertoire was obtained by high-throughput single-cell V(D)J sequencing after PCR amplification of porcine BCR genes. The clones of the IgG isotype were selected from O/18074-specific BCRs repertoire, and then, the expression and purification of pig-derived mAbs were performed as previously described (22). The purity and size of porcine mAbs were evaluated by reduced SDS-PAGE.

### Indirect immunofluorescence assay (IFA)

Baby hamster kidney-21 (BHK-21) cells in 24-well plates were infected with representative strains of FMDV serotypes A and O at a multiplicity of infection (MOI) of 1 and then washed with PBS and subsequently fixed in precooled methanol–acetone solution (ratio 1: 1, vol/vol) for 15 min at 4°C before the appearance of cell lesions. After washing with PBS, the cells were incubated with porcine mAb PO18-10 at a concentration of 5 µg/mL in a 200 µL volume for 1 h at 37°C. Subsequently, the cells were washed with PBS and incubated in the dark with fluorescein isothiocyanate (FITC)-labeled rabbit anti-porcine IgG (Thermofisher, USA) at a dilution of 1:1,000 for 1 h at 37°C. After three washes with PBS, the results were observed under the EVOS FL Imaging System (Life Technology, USA).

### SDS-PAGE and western blotting

FMDV serotypes O and A 146S antigens were denatured and reduced by heating at 95°C for 5 min in SDS-loading buffer and dithiothreitol (DTT). The samples were subjected to 12% SDS-PAGE and transferred to a methanol-activated nitrocellulose membrane followed by blocking with 5% non-fat milk in Tris-buffered saline with Tween 20 (TBST) overnight at 4°C. For native PAGE, FMDV serotypes O and A 146S antigens were mixed with loading buffer without SDS and DTT, and the samples were subjected to 10% native PAGE and transferred to a methanol-activated PVDF membrane followed by blocking with 5% non-fat milk in TBST overnight at 4°C. The membrane was incubated with porcine mAb PO18-10 (diluted to 2 µg/mL in PBS) and horseradish peroxidase (HRP)-conjugated rabbit anti-porcine IgG (Sigma, diluted 1/4,000 in PBS) in sequence for 1 h at 37°C. After a thorough wash with TBST, the membrane was incubated with enhanced chemiluminescence solution (Thermo Fisher Scientific, USA) for 1 min and subsequently exposed to X-ray film.

### Virus neutralization test

The porcine mAb PO18-10 was titrated for viral neutralizing activity against FMDV by using a micro-neutralization assay on monolayers of BHK-21 cells with representative FMDV O/18074 strain (Cathay topotype), O/GSLX/2010 strain (SEA topotype), O/HNNY/2022 strain (SEA topotype), O/Tibet/99 strain (ME-SA topotype), A/WH/CHA/09 (G1 subset of SEA97 lineage in ASIA topotype), A/GDMM/2013 strains (G2 subset of SEA97 lineage in ASIA topotype), and A/AF72 (A22 lineage in ASIA topotype). Briefly, 100 tissue culture infective doses (TCID_50_) of the virus were incubated with 2-fold serial dilutions of antibody in a 96-well plate at 37°C for 1 h, then 5 × 10^4^ BHK-21 cells/well were added to the plate as indicators of residual infectivity. Healthy cell and virus (0.1, 1, 10, and 100 TCID_50_) controls were used in duplicate per plate. The plates were incubated at 37°C under 5% CO2 conditions for 48 h before observing cytopathic effect (CPE). The acceptance criteria were set as complete CPE for 100 TCID_50_ virus control and no CPE for 0.1 TCID_50_ control. All live FMDV-related experiments were performed in a biosafety level (BSL)−3 laboratory at LVRI (China). The virus neutralization (VN) titer was expressed as the final antibody concentration to neutralize 100 TCID_50_ FMDV in 50% of the wells. A VN titer of 50 µg/mL was used as a cutoff for neutralization, and more than 50 µg/mL was considered a non-neutralization activity.

Neutralizing antibodies in serum samples were also detected using a VNT on BHK-21 cells, and the endpoint titers were calculated as the reciprocal of the last serum dilution to neutralize 100 TCID_50_ FMDV in 50% of the wells. The cutoff used in the VNT (1.65 log10) was based on the standard operating procedures of the World Reference Laboratory for FMD (WRLFMD, at the Pirbright Institute, United Kingdom).

### Biotinylation of mAb PO18-10

The high-purity mAb PO18-10 was conjugated with biotin using an EZ-Link Sulfo-NHS-LC-biotin reagent (Thermo Fisher Scientific, USA) according to the manufacturer’s instructions, and the resulting biotinylated mAb PO18-10 was named Bio-PO18-10.

### Development of C-ELISA

The optimum concentrations of the capture antibody (E32), 146S antigen (O/Mya/98), detection antibody (Bio-PO18-10), and horseradish peroxidase (HRP)-streptavidin were determined by checkerboard titration, and the incubation time and blocking buffer were optimized on the basis of the ratio between the reading values of negative and positive sera (N/P). C-ELISA was carried out under optimal conditions. Briefly, the purified mAb E32 diluted in carbonate-bicarbonate buffer (pH 9.6) was coated onto ELISA plate (Costar) at a concentration of 0.5 µg/mL in a 100 µL volume and incubated overnight at 4°C. The ELISA plate was washed three times with PBST (PBS with 0.1% Tween), and then, 100 µL of 1 µg/mL 146S antigen diluted in PBS was captured at room temperature for 2 h and washed three times with PBST. Subsequently, the ELISA plate was blocked with 100 µL blocking buffer (5% sucrose and 1% bovine serum albumin [BSA] in PBS) at 37°C for 60 min and washed three times with PBST. Serum samples were 2-fold serially diluted in 50 µL PBS (1:4–1:512) in duplicate and incubated with an equal volume of 1 µg/mL Bio-PO18-10 diluted in PBS at 37°C for 1 h. After five washes with PBST, 100 µL of 1:30,000-diluted HRP-conjugated streptavidin (GenScript, Inc.) was added, and the plates were incubated at 37°C for 15 min. After five washes with PBST, 100 µL of tetramethylbenzidine (TMB) was added to each well, and then, the mixture was incubated at 37°C for 10–15 min. The color reaction was terminated by the addition of 100 µL of 2M H_2_SO_4_, and the optical density (OD) readings were measured using an automatic microplate reader (BioTek) at a wavelength of 450 nm.

The FMDV-positive and -negative sera were tested simultaneously as internal standards in each ELISA plate. Four wells were used for antigen control (100% reactivity), and two wells were used as reaction blanks without 146S antigen and without serum. Antibody titers were expressed as the reciprocal (log10) of serum dilutions that provided 50% of the absorbance recorded in the antigen control wells (OD_50%_).

### Statistical analysis

Pearson’s coefficient test was used to determine the correlation between the C-ELISA titers and VNT titers, with *P*＜ 0.05 considered statistically significant.

Receiver operating characteristic curve (ROC) analysis (24) was used to calculate the assay cutoff value, sensitivity, and specificity by using 220 known negative serum samples (sera ⅰ) and 106 known positive serum samples (sera ⅱ).

GraphPad Prism software version 8.0 was used for the statistical analysis of the data.

## RESULTS

### Production and characterization of the mAb PO18-10

**Fig 1:**
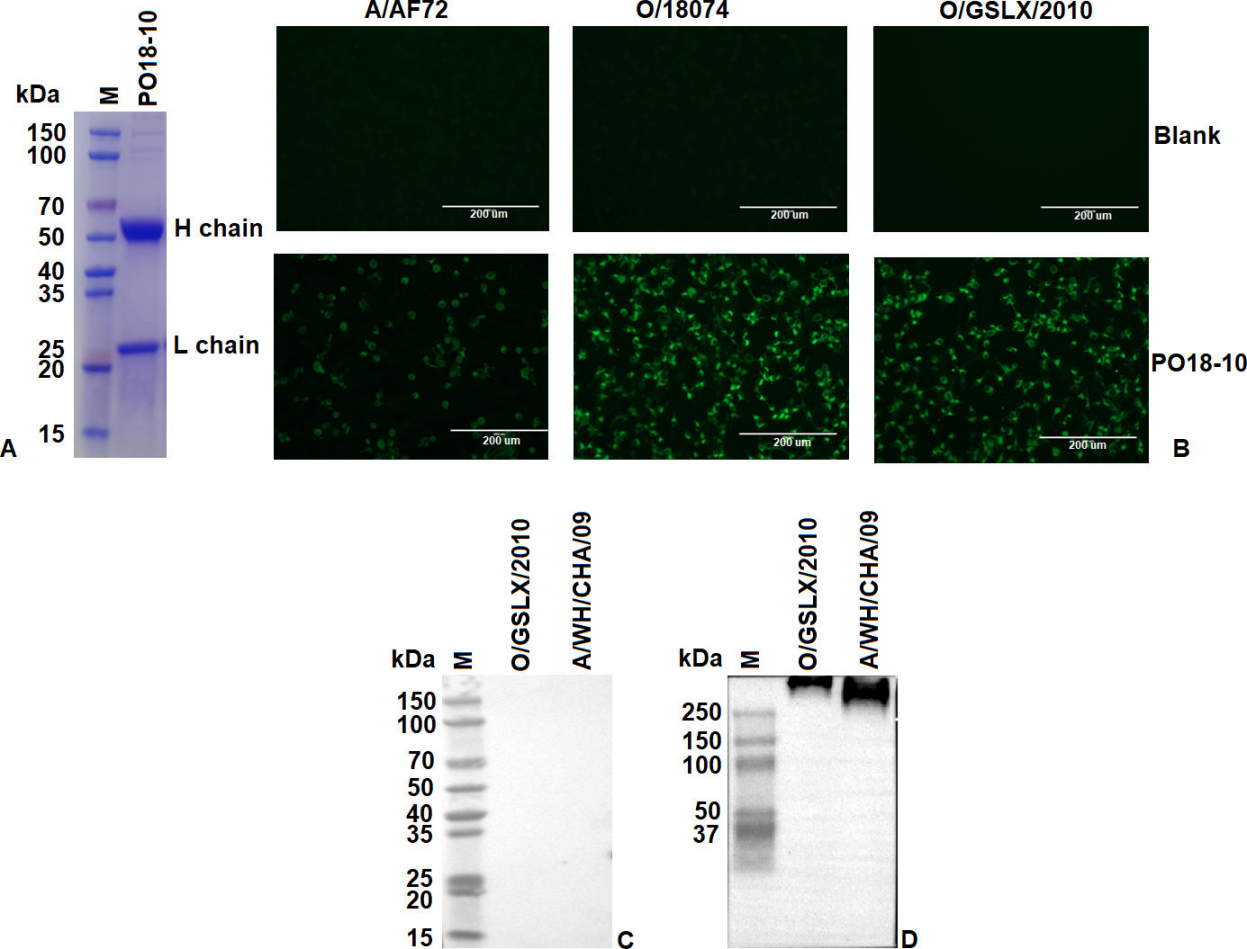
Production and characterization of the mAb PO18-10. (**A**) Expression analysis of the mAb PO18-10 by reduced SDS-PAGE. Lane M, protein molecular size markers (kDa). (**B**) Biological activity analysis of the mAb PO18-10 by IFA. IFA was performed using BHK21 cells infected with FMDV strain O/18074, O/GSLX/2010, or A/AF72 at an MOI of 1, fixed, and probed with 5 mg/mL of the mAb PO18-10, followed by incubation with rabbit anti-porcine IgG labeled with FITC (diluted 1:5,000 in PBS). (**C**) Western blotting identifies the reactivity of mAb PO18-10 with the denatured 146S antigens of FMDV O/GSLX/2010 and A/WH/CHA/09. (**D**) Western blotting identifying the reactivity of mAb PO18-10 with the native 146S antigens of FMDV O/GSLX/2010 and A/WH/CHA/09.

The porcine-derived mAb PO18-10 was successfully expressed in CHO cells, and purified mAb was analyzed by reduced SDS-PAGE. Two protein bands of approximately 54.6 kDa and 27.9 kDa were observed, which were consistent with the expected heavy chain and light chain of mAb PO18-10 (Fig. 1A). The biological activity against FMDV was determined using IFA. As shown in Fig. 1B, mAb PO18-10 exhibited binding affinity toward both FMDV serotypes O (O/18074 and O/GSLX/2010 isolates) and A (A/AF72 isolate).

To elucidate the characteristics of the epitopes recognized by mAb PO18-10, we conducted a WB assay using both denatured and intact FMDV 146S antigens to distinguish between linear or conformational antigen epitopes. For both serotypes O and A, the mAb PO18-10 did not bind to denatured viral antigens (Fig. 1C) but could bind to intact viral antigens (Fig. 1D), indicating recognition of conformational antigen epitopes on FMDV (25, 26).

### MAb PO18-10 exhibited broadly neutralizing activity against FMDV serotype A and serotype O

The viral neutralization titer and width of mAb PO18-10 were evaluated using VNT on BHK-21 with four representative FMDV serotype O viruses within three topotypes and three FMDV serotype A strains. As shown in Table 1, mAb PO18-10 showed neutralizing activity against four tested epidemic strains from serotype O and three representative strains from serotype A, indicating that mAb PO18-10 was an inter-serotype broadly neutralizing antibody.

**TABLE 1:** Neutralization activities of mAb PO18-10 against representative FMDV strains

FMDV serotypes	FMDV strains (Topotypes)	VN titers (µg/mL)^*a*^
Serotype O	O/18074 (CATHAY topotype)	4.43
	O/GSLX/2010 (SEA topotype)	5.57
	O/HNNY/2022 (SEA topotype)	21.18
	O/Tibet/99 (ME-SA topotype)	21.43
Serotype A	A/WH/CHA/09	3.00
	A/GDMM/2013 A/AF72	6.54 32

^*a*^ The VN titer was determined as the lowest antibody concentration that inhibits 50% of CPE.

### Determination of the cutoff value, sensitivity, and specificity of the C-ELISA

**Fig 2:**
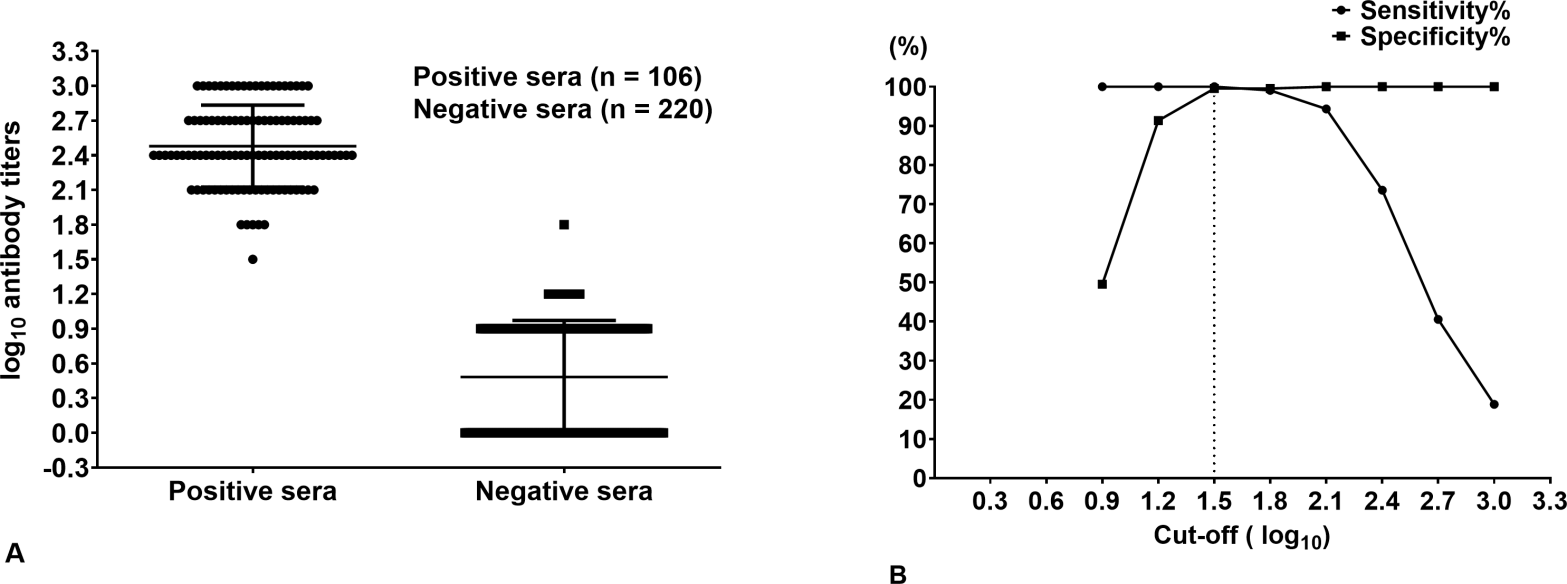
ROC analysis for the evaluation of the cutoff value, sensitivity, and specificity of the C-ELISA. (**A**) Dispersion of individual titers in the serum of naive animals (*n* = 220) and infected/vaccinated animals (*n* = 106). Antibody titers below the assay sensitivity (0.9 log10) are considered zero. (**B**) Values of the sensitivity and specificity of the C-ELISA were determined at various cutoff values. The dashed line indicates the selected cutoff value.

A total of 106 positive sera and 220 negative sera were tested using C-ELISA to determine the assay cutoff value, sensitivity, and specificity. The antibody titers of individual animals are shown in Fig. 2A. According to ROC analysis, when the cutoff value of C-ELISA was 1.5 log_10_, the values of sensitivity and specificity were optimal, and the sensitivity and specificity were 100% and 99.55%, respectively (Fig. 2B.). The area under the ROC was 0.9998 (standard error = 0.0001926), with a 95% CI of 0.9995–1.000. To improve the specificity, the antibody titer of 1.5 log_10_ was set as negative. Therefore, samples with titers ≤ 1.5 log_10_ were considered negative, and samples with titers > 1.5 log_10_ were considered positive.

### The cross-reactivity of C-ELISA with other viruses and other serotypes of FMDV

**Fig 3:**
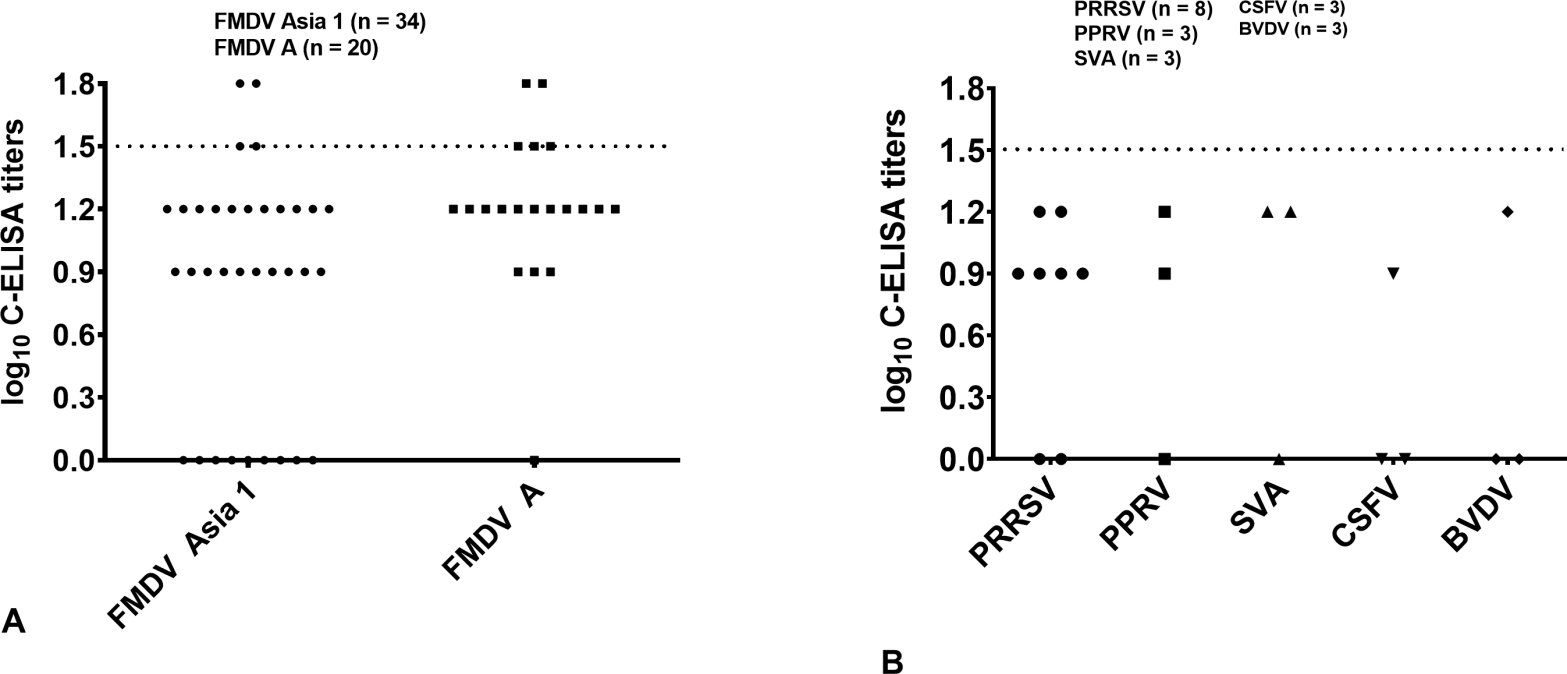
Cross-reactivity of C-ELISA with other viruses and FMDV serotypes Asia 1 and A. (**A**) Dispersion of individual titers of FMDV serotype Asia 1-positive sera (*n* = 34) and A-positive sera (*n* = 20). (**B**) Dispersion of individual titers of PRRSV-positive sera (*n* = 8), CSFV-positive sera (*n* = 3), SVA-positive sera (*n* = 3), BVDV-positive sera (*n* = 3), and PPRV-positive sera (*n* = 3). Antibody titers below the assay sensitivity (0.9 log10) are considered zero. The dashed line indicates the cutoff value.

The cross-reactivity with other serotypes of FMDV was evaluated using 20 sera positive for serotype A and 34 sera positive for serotype Asia 1 (serum sample set V). As shown in Fig. 3A, two of 34 serotype Asia 1-positive sera and two of 20 serotype A-positive sera showed positive results when tested by C-ELISA, indicating that there is a weak cross-reaction with sera positive for FMDV serotypes A and Asia 1.

The evaluation of the cross-reaction of C-ELISA with sera from other virus-infected animals (SVA, CSFV, PRRSV, PPRV, and BVDV) indicated that there was no cross-reaction with other virus-infected sera (Fig. 3B).

### Comparison of results obtained with C-ELISA and VNT

**Fig 4:**
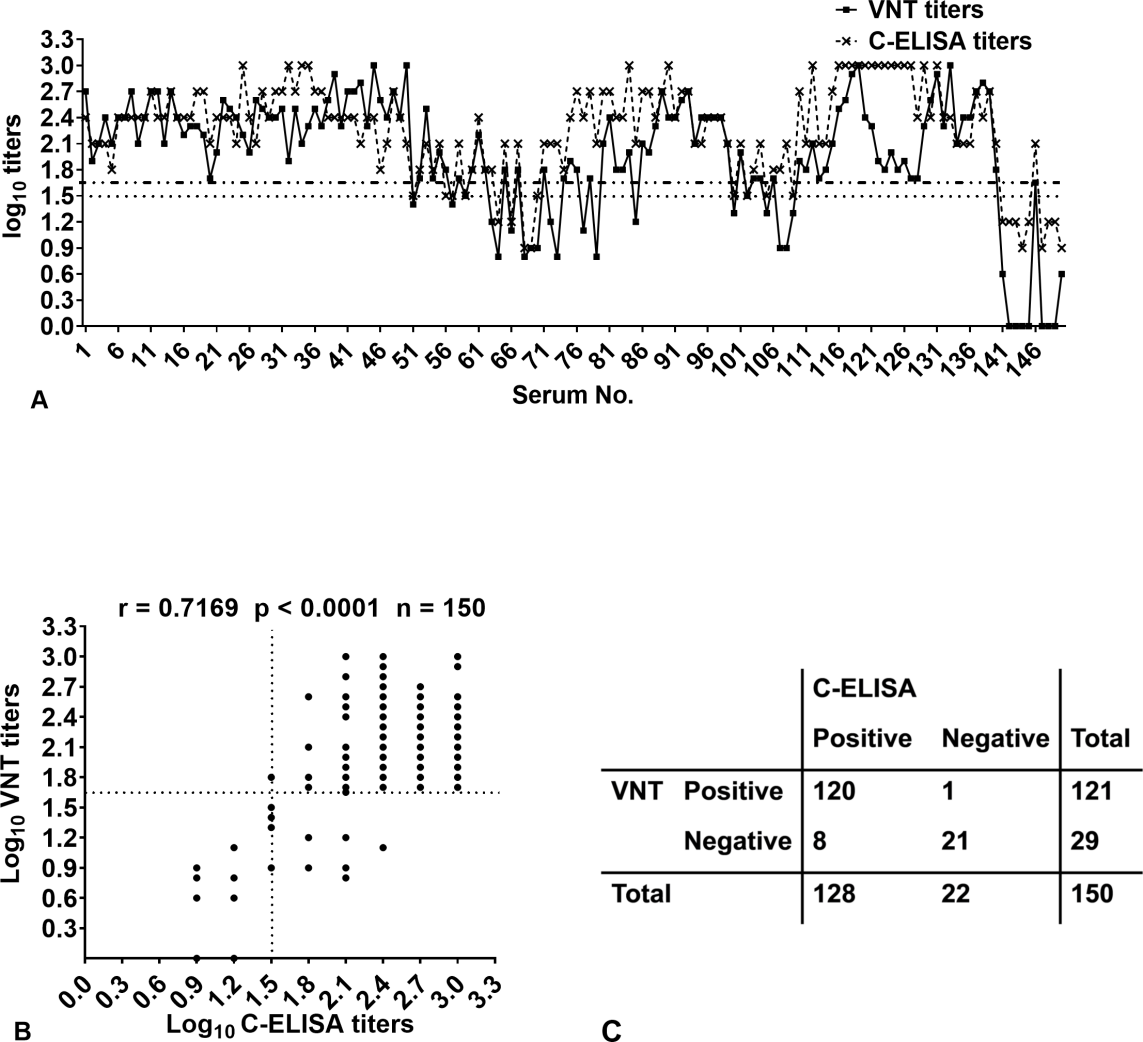
Correlation between C-ELISA titers and VNT titers. (**A**) Dispersion of individual values obtained using VNT and C-ELISA. The dashed lines, ····· and -··-··, represent the cutoff values of C-ELISA and VNT, respectively. (**B**) Correlation between antibody titers as measured by VNT and C-ELISA. n, number of sera tested, r, correlation coefficient. The *P* value is two-tailed. The dashed lines represent the cutoff values. Note: some dots represent more than one serum sample. (**C**) The two-by-two table indicates the positive/negative relationship between the C-ELISA and VNT. Serum with a titer >1.5 log10 and a titer ≥1.65 log10 was considered positive for C-ELISA and VNT, respectively.

To assess the validation of C-ELISA for detecting neutralizing antibodies against FMDV serotype O, 150 serum samples with varied titers of positive antibodies against FMDV serotype O or negative antibodies were tested by C-ELISA, and the results were compared with those obtained by VNT. The antibody titers of individual animals are shown in Fig. 4A, and the positive/negative results are summarized in Fig. 4C. A total of eight serum samples (sera no. 63, 72, 73, 77, 79, 85, 107, and 108) were positive for C-ELISA and negative for VNT. Only one serum (serum no. 56) tested negative for C-ELISA and positive for VNT. The results of the remaining 141 serum samples were completely consistent between the two methods. The coincidence rate of the positive/negative results of the C-ELISA and VNT was 94% (141/150). The Pearson correlation coefficient between C-ELISA titers and VNT titers was calculated by comparing the data at the individual level, and a good correlation with an r value of 0.7169 (*P* < 0.0001) was observed (Fig. 4B).

### Post-vaccination kinetics of neutralizing antibody response

**Fig 5:**
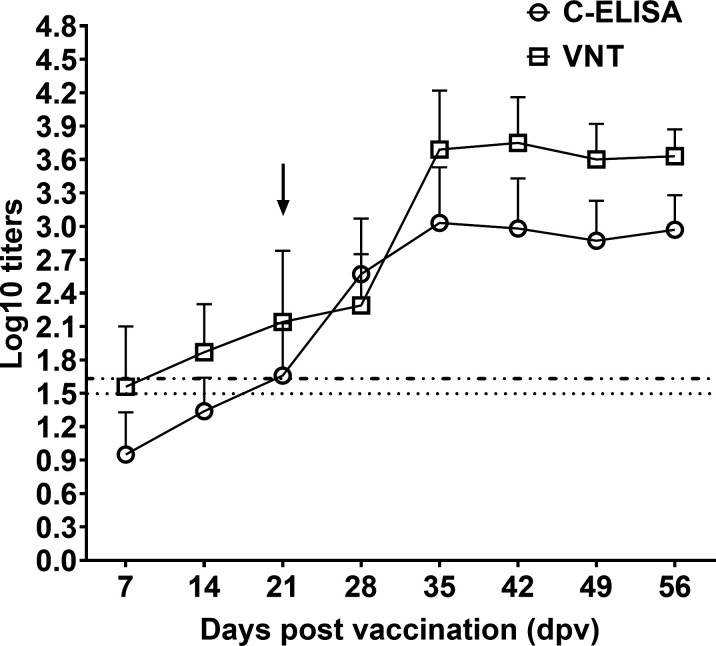
Neutralizing antibody dynamics of sera from 20 pigs vaccinated twice with FMDV O/Mya/98 inactivated vaccine analyzed by C-ELISA and VNT. Serum samples were collected at 7, 14, 21, 28, 35, 42, 49, and 56 days post-vaccination. The arrow represents booster vaccination at the given time; the dashed lines, ····· and -··-··, represent the cutoff values of C-ELISA and VNT, respectively. Data represent the mean + SD.

To analyze neutralizing antibody dynamics, endpoint titers of sera collected weekly from 20 pigs vaccinated with FMDV O/Mya/98 inactivated vaccine on days 0 and 21 were determined both by C-ELISA and VNT. As shown in Fig. 5, in the case of C-ELISA, pigs produced detectable neutralizing antibodies at 7 dpv, and the titers gradually increased, reaching the maximum value at 35 dpv (2 weeks post-booster vaccination). Subsequently, it remained stable until the last blood collection at 56 dpv. The kinetics of the antibody response determined by C-ELISA was relatively similar to that determined by VNT.

### Assessment of the feasibility of detecting heterologous antibodies

**Fig 6:**
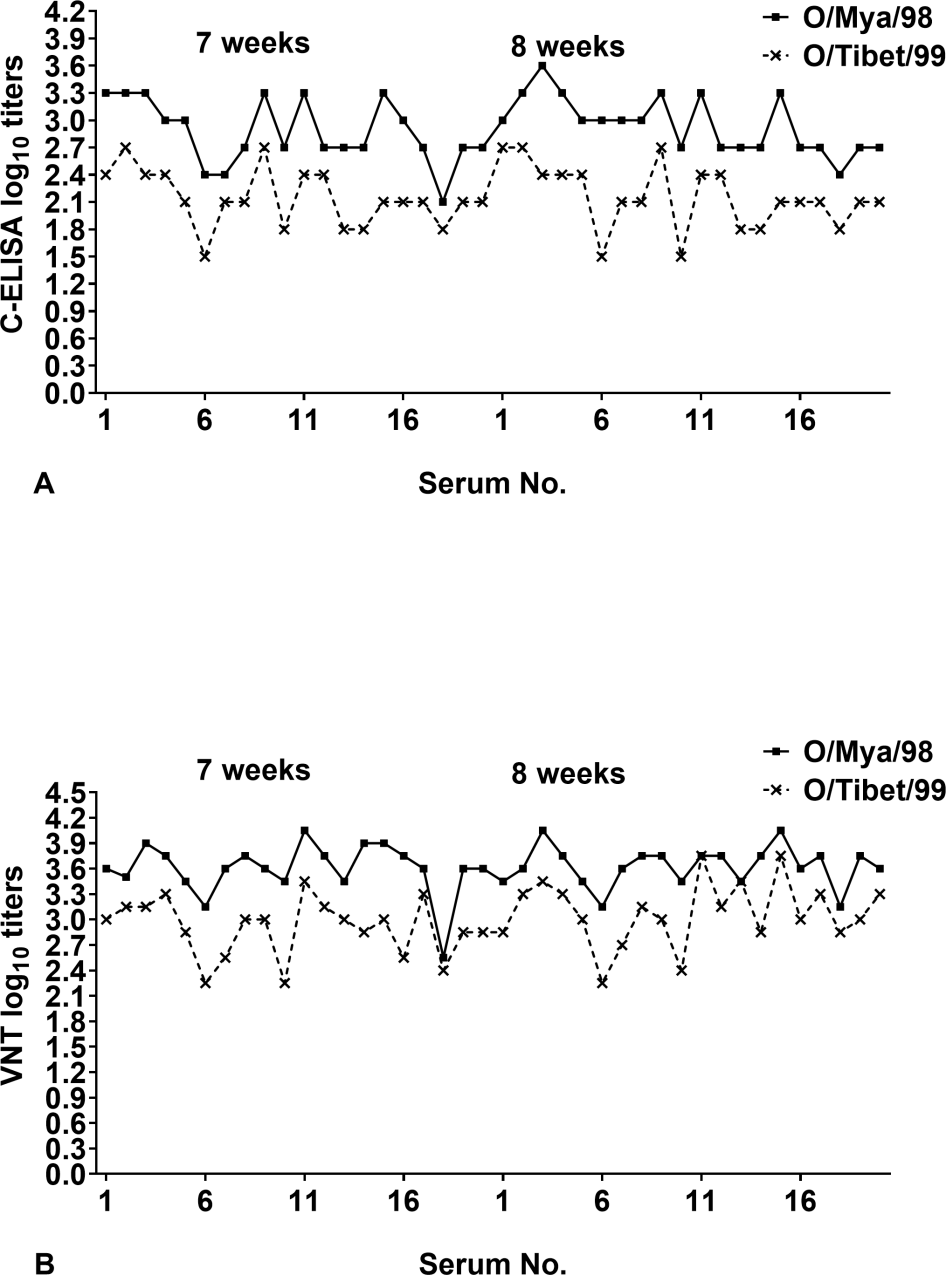
Comparison between heterologous antibody and homologous antibody. A total of 40 sera pooled from 20 pigs inoculated with inactivated FMDV O/Mya/98 vaccine at 7 and 8 weeks post-vaccination was measured by C-ELISA (**A**) and by VNT (**B**) for the FMDV O/Mya/98 (homologous strain) and FMDV O/Tibet/99 (heterologous strain).

Endpoint titers of sera collected from 20 pigs inoculated with inactivated FMDV O/Mya/98 vaccine at 7 and 8 weeks post-vaccination were detected by C-ELISA and VNT using both FMDV O/Mya/98 and FMDV O/Tibet/99 strains as antigen. As shown in Fig. 6A, the titer of homologous antibody (FMDV O/Mya/98) was higher than that of heterologous antibody (FMDV O/Tibet/99) in each animal by C-ELISA. This result was consistent with the results obtained by VNT (Fig. 6B).

## DISCUSSION

In this study, a mAb named PO18-10 was successfully isolated using single-B-cell isolation techniques from the PBMCs of experimentally vaccinated pigs and characterized using IFA, WB, and VNT. MAb PO18-10 reacted with both FMDV serotypes O and A strains in IFA and showed broadly neutralizing activity against representative strains of FMDV serotype A and serotype O in VNT, indicating that mAb PO18-10 was an inter-serotype broadly neutralizing antibody. When it was tested for WB of capsid protein, it failed to bind with denatured FMDV 146S antigen but could bind to the native 146S antigen. This result indicates that it may recognize conformational epitopes (25, 26). Work is underway to identify epitopes by selecting mAb neutralization-resistant mutants (27), resolving the cryo-electron microscopy structure of the antigen-antibody complex, and rescuing site-directed FMDV mutants by reverse genetics (28–30), which will be described in detail in the following paper. Previously, a fully bovine-derived mAb W145 with broadly neutralizing activity against FMDV serotype A was isolated and used as a detector antibody to develop a competitive ELISA for the detection of FMDV serotype A-specific neutralizing antibodies (23). Here, a competitive ELISA method based on a porcine-derived inter-serotype broadly neutralizing mAb PO18-10 for detecting FMDV serotype O neutralizing antibodies was developed. FMDV serotype O is one of the most prevalent serotypes in the world, and it is also the major cause of FMD outbreaks in China. It is of great significance to develop a novel C-ELISA based on natural host-derived mAb as a more reliable tool for evaluating herd immunity and ultimately contributing to control the FMDV spread in the world.

The cross-reactivity of C-ELISA with sera positive for other circulating viruses in cattle, sheep, and pigs was tested using 20 sera from PRRSV-infected pigs (*n* = 8), SVA-infected pigs (*n* = 3), CSFV-infected pigs (*n* = 3), PPRV-infected sheep (*n* = 3), and BVDV-infected cattle (*n* = 3), and no cross-reaction was observed. When C-ELISA was used to test sera positive for FMDV serotype A and serotype Asia 1, two of 34 Asia 1-positive sera and two of 20 A-positive sera were positive. The four positive sera were all from animals immunized three times with inactivated FMDV Asia 1 or FMDV A vaccines. Investigation of these four sera using VNT showed that all four sera were also positive (data not shown), indicating that this cross-reaction between serotypes was caused by heterotypic humoral immune responses rather than non-specific reactions. Historical data have shown that African buffalo infected in the field with FMDV serotypes SAT1, SAT2, or SAT3 produced high titers of neutralizing antibodies against FMDV serotypes A, O, C, and Asia 1, a virus serotype that has never been recorded in Africa (31). A more recent study also showed that the LPB-ELISA titers specific for FMDV serotype C significantly increased following sequential vaccination with inactivated FMDV serotypes O, A, and Asia 1, and in the absence of vaccination with inactivated FMDV serotype C antigen. These animals had clearly developed a cross-reactive FMDV-specific response (32).

To obtain broadly neutralizing FMDV monoclonal antibodies, pigs were sequentially vaccinated with different topotypes of FMDV in different serotypes. Because broadly neutralizing antibodies generally target conserved neutralizing epitopes, these conserved epitopes often exhibit weak immunogenicity and do not produce antibodies easily. As a result, continuous stimulation with a variety of antigen molecules is required to generate the accumulation of mutations in the complementarity-determining region (CDR) of antibodies, ultimately leading to the production of broadly neutralizing antibodies (33). With this heterologous sequential vaccination regime, we previously obtained the FMDV serotype O-specific broadly neutralizing monoclonal antibodies (21) and serotype A-specific broadly neutralizing monoclonal antibodies (34). The results of this study also showed that an inter-serotype broadly neutralizing monoclonal antibody PO18-10 was successfully obtained from sequentially vaccinated pigs. The protocol described in this study for generating porcine monoclonal antibodies against FMDV is applicable for isolating porcine monoclonal antibodies against other porcine viruses, such as SVA (35), CSFV, and PRRSV. MAb PO18-10 isolated in this study showed good performance in a competitive ELISA for detecting neutralizing antibodies against FMDV serotypes O; the specificity and sensitivity of the assay were evaluated to be 99.55% and 100%, respectively. Additionally, C-ELISA developed here is capable of detecting heterologous antibodies, but the titer of heterologous antibodies is lower than that of homologous antibodies, which is in line with the results of VNT. Hence, for the purpose of accurately assessing the efficacy of the vaccine, 146S antigen matching the vaccine strain should be used.

In summary, a fully porcine-derived mAb PO18-10 with inter-serotype broadly neutralizing activity against FMDV serotype A and serotype O was successfully obtained by single-B-cell antibody techniques. A competitive ELISA based on this natural host-derived mAb for the detection of neutralizing antibodies against FMDV serotype O was developed and validated. The assay demonstrates high sensitivity, specificity, and coincidence rate with VNT, making it a reliable tool for confirming FMDV infection and evaluating the vaccine efficacy.

## Data Availability

The sequences of mAbs E32 (GenBank accession numbers: MN612733.1, MN612678.1) and PO18-10 (Chinese Patent Application number: 202410612113.0) are available from the corresponding author upon reasonable request.
